# The Symmetry of Neural Stem Cell and Progenitor Divisions in the Vertebrate Brain

**DOI:** 10.3389/fcell.2022.885269

**Published:** 2022-05-25

**Authors:** Glòria Casas Gimeno, Judith T. M. L. Paridaen

**Affiliations:** European Research Institute for the Biology of Ageing (ERIBA), University Medical Center Groningen (UMCG), University of Groningen, Groningen, Netherlands

**Keywords:** Neurogenesis, neural stem cell, asymmetric division, brain development, cellular asymmetries, radial glial cells, symmetry-breaking

## Abstract

Robust brain development requires the tight coordination between tissue growth, neuronal differentiation and stem cell maintenance. To achieve this, neural stem cells need to balance symmetric proliferative and terminal divisions with asymmetric divisions. In recent years, the unequal distribution of certain cellular components in mitosis has emerged as a key mechanism to regulate the symmetry of division, and the determination of equal and unequal sister cell fates. Examples of such components include polarity proteins, signaling components, and cellular structures such as endosomes and centrosomes. In several types of neural stem cells, these factors show specific patterns of inheritance that correlate to specific cell fates, albeit the underlying mechanism and the potential causal relationship is not always understood. Here, we review these examples of cellular neural stem and progenitor cell asymmetries and will discuss how they fit into our current understanding of neural stem cell function in neurogenesis in developing and adult brains. We will focus mainly on the vertebrate brain, though we will incorporate relevant examples from invertebrate organisms as well. In particular, we will highlight recent advances in our understanding of the complexities related cellular asymmetries in determining division mode outcomes, and how these mechanisms are spatiotemporally regulated to match the different needs for proliferation and differentiation as the brain forms.

## 1 Introduction

In central nervous system (CNS) development, pluripotent neural precursors derived from the ectoderm are responsible for the production of all types of neurons and macroglial cells, as well as adult progenitor cells. In vertebrates, neural stem and progenitor cells (collectively named neural progenitors, abbreviated as NPCs) arise from the neuroepithelium that lines the nascent neural tube. As typical epithelial cells, neural progenitors exhibit well-defined apicobasal polarity, with their apical side facing the internal lumen of the neural tube and their basal membrane contacting the pial surface. The neuroepithelium appears as a pseudostratified epithelium, with cell nuclei distributed along the entire apicobasal axis. NPCs exhibit interkinetic nuclear migration, a stereotyped movement of the nucleus towards the apical surface in the G_2_ phase of the cell cycle, ensuring that NPC mitosis occurs at the ventricular surface.

At early stages prior to the onset of neurogenesis, self-renewing cell divisions expand the NPC pool. After the onset of neurogenesis, NPCs start producing neurons that migrate basally and start populating upper layers of the tissue. In mid-neurogenic stages, in some parts of the brain, especially in expanded regions such as the neocortex in mammals, a diverse range of specialized intermediate progenitors are generated from asymmetrically dividing NPCs termed radial glial cells (RGCs). Newborn basal progenitors (BPs) delaminate from the ventricular surface and migrate to a more basally located germinal zone, the subventricular zone. Depending on the species, these BPs have low or high self-renewing capacity and serve to increase neuronal production from one initial RGC (Sections 5.3 and 5.4 for more details). At the end of embryonic neurogenesis, NPCs switch to gliogenesis and produce astrocytes, oligodendrocytes and ependymal cells.

As in any developing tissue, the timing of stem cell proliferation and differentiation needs to be tightly regulated in order to accommodate tissue growth and maturation in changing spatial constraints. Moreover, while the neural tube starts off as a relatively homogeneous structure, the mature CNS is composed of structurally and functionally distinct regions with different cellular composition. Therefore, the ratio between proliferation and differentiation needs to be regulated also at the local level to allow for these regional specializations. In this review, we will discuss the mechanisms that underlie the balanced ratio between self-renewal and differentiation in the context of division mode regulation.

## 2 Neural Progenitor Cell Division Mode and Division Outcomes

**FIGURE 1:**
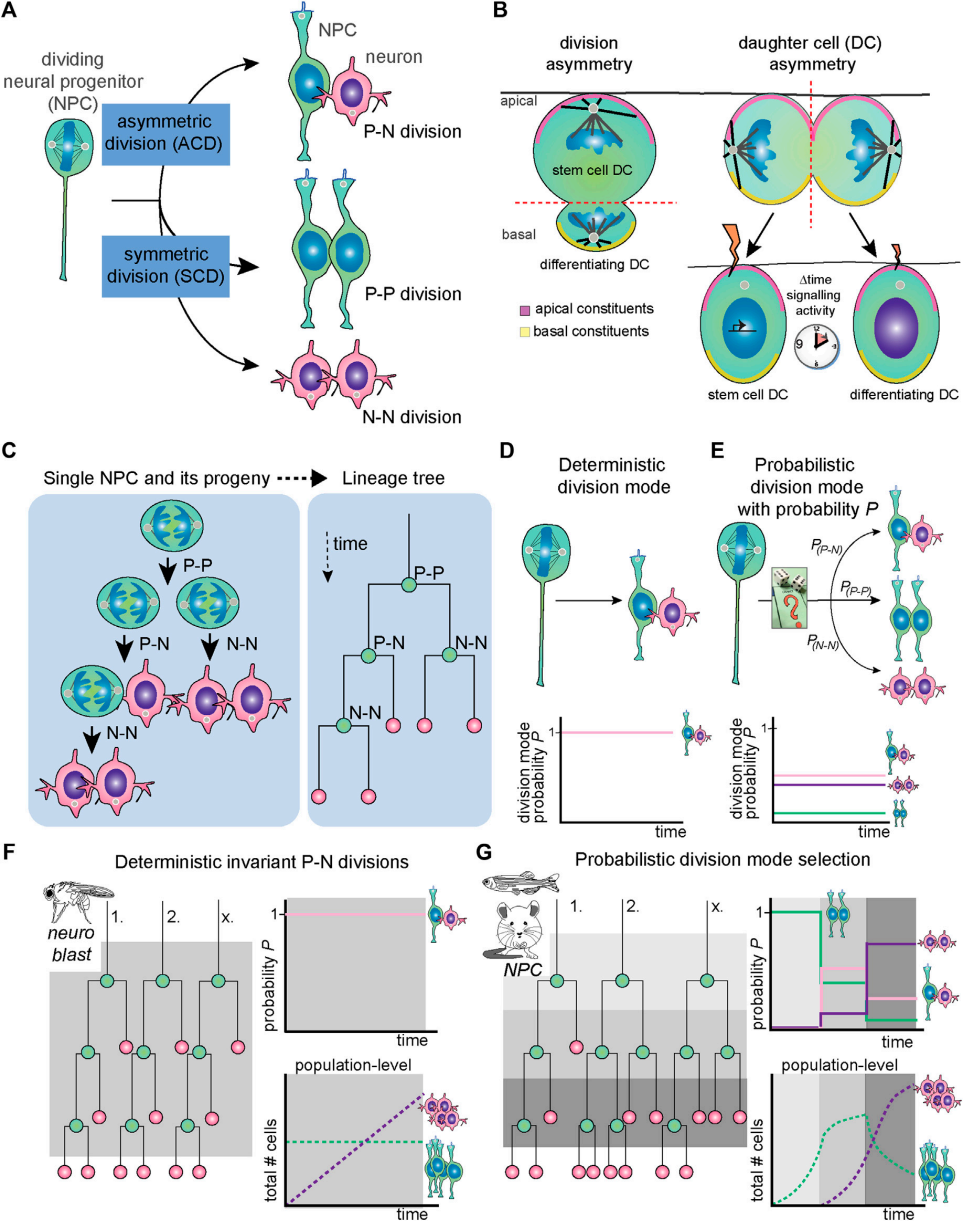
General concepts regarding NPC division mode, mechanism of asymmetry and deterministic versus probabilistic division modes. **(A)** As any stem cell type, NPCs can undergo asymmetric division [typical example is a progenitor-neuron (P-N) division], symmetric proliferative division (P-P division) or symmetric differentiative (N-N) division. **(B)** In division asymmetry (left), asymmetrical daughter cell fates are induced by morphological asymmetries in terms of cell size, cleavage furrow orientation, apicobasal polarity and unequal distribution of fate-determinants. In daughter cell asymmetry (right), the division itself is morphologically symmetrical. However, small fluctuations in signaling states due to differential inheritance of signaling components and/or stochastic fluctuations in transcriptional activity lead to unequal and/or asynchronous signaling activity, which ultimately induces two unequal daughter cell fates. **(C)** The progeny of one single NPC and division modes of each round of cell division is shown (left) and translated into a lineage tree (right). **(D)** Deterministic division mode is defined as having a probability of a specific division mode (in this example, P-N division) of 1. **(E)** In probabilistic division mode, there are specific probabilities for each type of division mode named *P*_*P-N*_, *P*_*P-P*_ and *P*_*N-N*_. **(F)** In systems with deterministic and invariant asymmetric divisions, such as the *Drosophila* neuroblast, the *P*_*P-N*_ remains stable over time (bottom panel), whereas in probabilistic division, *P*_*P-N*_, *P*_*P-P*_ and *P*_*N-N*_ can assume different values depending on the context. **(G)** In systems with probabilistic division mode selection, such as mouse and zebrafish NPCs, the probabilities *P*_*P-N*_, *P*_*P-P*_ and *P*_*N-N*_ change over developmental time so that the predominant division mode in individual NPCs shifts. Thus, regulation of the spatiotemporal balance between proliferation and differentiation ensures robust development at the population level. DC, daughter cell; N, neuron; NPC, neural progenitor cell; P, progenitor, *P*, probability.

A common biological strategy for mediating the balance between self-renewal and differentiation is the regulation of division modes (Figure 1A). In short, stem cell division can be proliferative, producing two new progenitor cells (a so-called P-P division); asymmetric, producing a new stem cell and a differentiating cell (P-N division); and terminal self-consuming, producing two cells initiating neuronal differentiation (N-N division). More generally, cell divisions can be considered asymmetric when they produce two different types of progenitors or two types of terminally differentiated cells.

Here, we can distinguish two concepts of mediating asymmetrically fated daughter cells, namely division asymmetry and daughter cell asymmetry (Figure 1B). In division asymmetry, asymmetries between daughter cells arise directly during cell division (Figure 1B, left panel). For instance, if division is such that during mitosis, subcellular structures are already asymmetrically partitioned between sister cells, or if sister cells have different sizes or display other morphological asymmetries. A classic example of division asymmetry is found in the *Drosophila* neuroblast, which has been extensively studied [Figure 1B, left; reviewed by (Loyer and Januschke, 2020)]. On the other hand, in daughter cell asymmetry, the mother cell splits in seemingly identical sister cells, and diverging fates arise sometime after division (Figure 1B, right panel). The cell division is symmetrical in the sense that morphologically, the cleavage of the mother cell is such that two equal sized and shaped daughter cells have emerged. However, through unequal exposure to signals, the daughter cells subsequently obtain different cell fates. Vertebrate NPC divisions that yield one progenitor and one differentiating daughter cell often portray this type of asymmetry. As we will see below in Sections 3 and 4, different mechanisms are used to result in asymmetric daughter cell fates arising from morphologically symmetric divisions.

The concepts of division asymmetry and daughter cell asymmetry overlap, as unequal segregation of intracellular parts leading to or biasing daughter cell fates could also be considered as division asymmetry. Moreover, it is likely that additional hidden asymmetries in segregation of subcellular content occur that contribute to daughter cell asymmetry despite having morphologically symmetrical divisions.

There are distinct molecular mechanisms by which asymmetric fates in sister cells can be introduced. These can be classified as intrinsic mechanisms, in which cell division results in two intrinsically different sister cells (e.g., asymmetric distribution of fate determinants in mitosis), or extrinsic, in which newborn sibling cells are virtually indistinguishable but lead to diverging fates by external influence (e.g., different exposure to extracellular signals). Some of these mechanisms appear to depend on initial small fluctuations due to stochastic processes such as transcription (see also below in Section 3). While many mechanisms of symmetry-breaking in neural stem cell division have been described, a *bona-fide* generally applicable fate determination mechanism or combination thereof does not seem to be the case, as we will discuss in the next section.

## 3 Stochasticity Versus Determinism in Division Mode Selection and Lineage Progression

At the tissue level, NPC division mode progresses from symmetric proliferative to asymmetric and symmetric neurogenic divisions as brain development proceeds (Figure 1C). However, division mode progression at the single NPC level seems to be more heterogeneous in vertebrates. Experimentally, the study of division mode selection is challenging because it requires long-term following of sister cells after cell division. However, lineage tracing and time-lapse imaging have provided insights in the pattern of division modes used by individual NPCs in different developing organisms.

In invertebrate organisms like *Drosophila*, neurogenesis results from NPCs divisions that follow a fixed pattern of subsequent divisions modes [Figures 1D,F; reviewed by (Loyer and Januschke, 2020)]. In this case, it could be noted that division mode and cell specification is invariant and underlying mechanisms deterministic in nature (Figure 1D; reviewed by Zechner et al., 2020). In contrast, studies of single NPC lineages in vertebrate systems show that individual clones follow a variety of trajectories and do not follow a strict pattern of division modes (Figures 1E,G). This was shown in the retina, where there is a stereotyped order of neuronal cell type birth. For instance, in the zebrafish and rat retina, tracking of individual clones through time-lapse imaging with cell fate markers shows high variability in the clonal size and composition of the lineages generated by individual NPCs (Figure 1G, Gomes et al., 2011; He et al., 2012). Similarly, live imaging of NPCs in the developing zebrafish telencephalon and hindbrain show heterogeneity in the NPC division modes present at neurogenic stages (Dong et al., 2012; Hevia et al., 2021). Intriguingly, in the zebrafish retina, the probabilities for retinal NPCs to undergo P-P, P-N or N-N divisions change over time (Figure 1G). These temporal changes ensure that at the tissue level, for each developmental stage the proper balance between proliferation and differentiation is achieved (Figure 1G).

Individual NPC division modes have also been investigated in developing mammalian brains. Here, it is difficult to track entire NPC lineages through live imaging. Instead, sparse labelling of individual NPCs and their progeny is achieved through low-titer retrovirus intraventricular injection and genetic tools, such as Mosaic Analysis with Double Markers (MADM). With these techniques, the lineages downstream of either both or one of the daughter cells arising from a division can be specifically traced (Gao et al., 2014; Llorca et al., 2019). Such single-clone tracing studies in the mouse cortex have reached somewhat contradicting results on individual NPC lineage generation (Gao et al., 2014; Llorca et al., 2019). Based on MADM tracing, the first study proposed that the neuronal output of individual NPCs in the cortex shows little heterogeneity and is quite predictable (Gao et al., 2014). Upon onset of neurogenesis, mouse NPCs were calculated to produce 8–9 neurons on average. Furthermore, about 1 in 6 NPCs were determined to proceed to gliogenesis upon finishing embryonic neurogenesis. In contrast, a more recent study showed higher diversity of clonal size and generated neuronal types per lineage (Llorca et al., 2019) similar to the earlier work in the vertebrate retina. In this study, sparse retroviral labelling, mosaic genetic Cre-lox based labelling as well as MADM were applied and results compared. A stochastic model with specific fixed probabilities for each division mode which that change over time fits the experimental observations well (Llorca et al., 2019). In this model, spatiotemporally regulated changes in probabilistic division mode and daughter cell fate selection by individual cells is key to building a reproducible pattern of neuronal layers and types in the mammalian forebrain (Figure 1G). While these different conclusions may seem difficult to reconcile at first glance, when technical restrictions such as the fact that MADM system only works in mitotic cells are considered, both studies are in agreement on the multipotency and average lineage sizes generated from the majority of mouse neocortex NPCs.

In this context, an important additional question is whether a subset of lineage-restricted NPCs, that is NPCs that are competent or biased to generate certain types of neurons, exists. Although data regarding this question is conflicting, taken together they suggest that at least in mammals, neurogenesis is mediated through multipotent NPCs as well as a small population of lineage-restricted NPCs that are biased to generate upper layer neurons (Llorca et al., 2019).

These findings suggest that non-determinism and apparent stochasticity (absence of predictableness) is an important factor in division mode selection in vertebrate brain development [more extensively reviewed by (Zechner et al., 2020)]. At the same time, many factors and processes have been described to influence cell division outcomes or to increase the probability of certain division outcomes (Figure 1E, see also below in Section 4). Biological processes such as transcription and molecular interactions between limited amounts of molecules are unpredictable and therefore stochastic by nature. Therefore, stochastic processes are proposed to contribute to the heterogeneity in division mode selection by individual NPCs [see also (Hiesinger and Hassan, 2018)]. However, mechanisms that are more deterministic and predictable are very relevant as well, as we will discuss in the next sections. Moreover, it is likely that specific factors or processes that result in division asymmetry, especially those that are technically challenging to visualize and measure, remain unknown and hidden. Taken together, it is probable that a weighted combination of deterministic factors and processes, stochastic fluctuations and biases, as well as still hidden asymmetries determine division mode used by individual NPCs. The relevant weight of each fate-determining factor and process, and exact combination used is likely to be stage-, species- and time-dependent.

## 4 Which NPC Properties Are Involved in Division Mode Selection?

**FIGURE 2:**
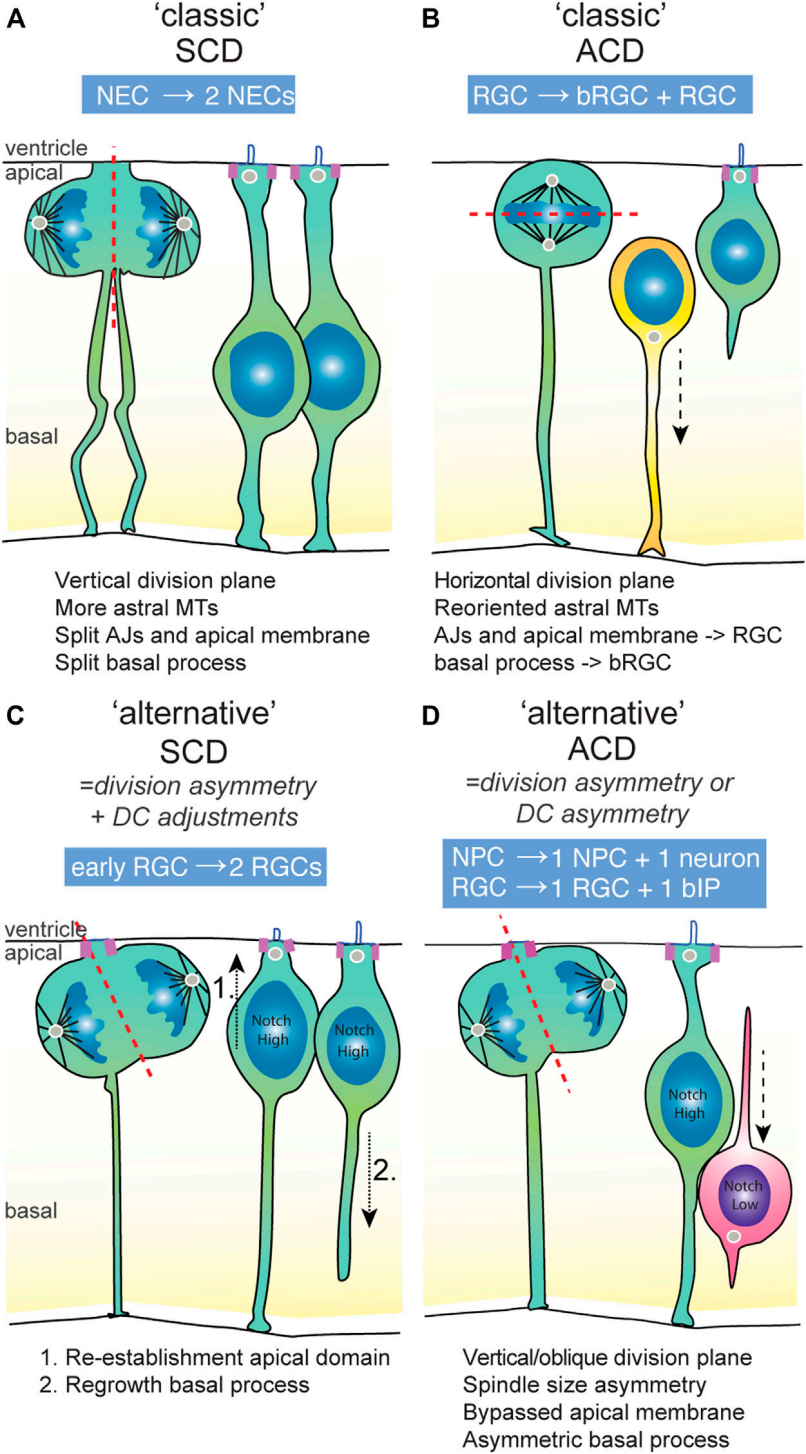
The symmetric and asymmetric division modes used in vertebrate developing brains. **(A)** An example of a classical division symmetry, namely the neuro-epithelial cell (NEC) that undergoes P-P division prior to the onset of neurogenesis. The division plane is vertical, which splits the AJs, apical domain and basal process into equal parts that are inherited by either daughter cell. The presence of astral microtubules limit wobbling of the spindle, ensuring division symmetry. **(B)** An example of a classical division asymmetry in the mammalian developing brain, namely the radial glial cell (RGC) that generates basal RGC in an asymmetric division. The division plan is horizontal due to the re-oriented spindle towards the apical domain. This results in unequal segregation of the apical and basal constituents to the RGC and bRGC daughter cell, respectively. **(C)** An example of an “alternative” symmetric division in early stages of mammalian neurogenesis, in which an initial morphological division asymmetry is compensated for by adjustments in the daughter cells eading to symmetric Notch signalling. These adjustments constitute re-establishment of the apical and basal domain by the non-inheriting daughter cell, ensuring daughter cell fate symmetry. **(D)** An example of an “alternative” asymmetric division, which is typical for most NPC divisions in the vertebrate brain. The division plane is mainly vertical or slightly oblique. Therefore, the division is partially asymmetric, though some parts of the cell (like the apical domain) appear to be equally bisected. In general, the basal process is inherited by the NPC daughter cell. Because of asymmetric Notch signalling states between the daughter cells, the other daughter cell becomes an basal intermediate progenitor (IP) or neuron. ACD, asymmetric cell division; AJs, adherens junctions; bIP, basal intermediate progenitor; bRGC, basal radial glia; DC, daughter cell; NEC, neuroepithelial cell; NPC, neural progenitor cell; RGC, radial glial cell; SCD, symmetric cell division.

In general, adoption of neuronal versus progenitor daughter fates is characterized by several aspects of their cell biology. First, newborn neurons typically need to lose their apical domain that tethers them to the ventricular surface in order to allow them to delaminate from the ventricular surface, initiate neuronal differentiation and move basally to their final position in the neuronal layer(s) [Figure 2D; reviewed by (Singh and Solecki, 2015)]. This loss of apical domain can occur through division asymmetry, in which the neuronal daughter either did not inherit the apical membrane, or loses it through downregulation of apical adhesion complexes or abscission of apical membrane after division. In many vertebrate systems including mice, zebrafish and chick, after a neurogenic asymmetric division, the apically positioned daughter cell inherits (part of) the apical domain and induces neuronal differentiation (Figure 2D). In contrast, in general, the more basally positioned progenitor daughters either retain or re-establish an apical domain containing adhesion and polarity complexes (Figures 2C,D). Second, NPCs that maintain “stemness” usually also retain or regrow the basal process that spans the width of the neuroepithelium basally (Figure 2C). These general cell biological properties coupled to progenitor or neuronal fate often play key roles in mechanisms underlying (a)symmetric division modes.

In the next sections, we will provide an overview of currently known aspects of cell division and NPC properties that have been demonstrated to influence their division mode in development of the vertebrate nervous system. Many of these NPC properties are related to their unique morphology and cell biology. Moreover, as we will discuss below in this section and in Section 5, these properties are also often connected in some way to the regulation of, or are being regulated by, the activity of signaling pathways. Delta-Notch signaling and Sonic Hedgehog (Shh) are known to be of particular importance in NPC proliferation and differentiation (Garcia et al., 2018; Moore and Alexandre, 2020). Other pathways that play a prominent role are the FGF, Wnt and Hippo signaling pathways. Together, this supports a model where signaling pathways are intricately connected to generate particular division outcomes.

It is important to note that experimental evidence is naturally often limited to certain regions of the CNS or specific cellular subtypes, and thus the extent to which these are part of a universal model of vertebrate NPC regulation, and even if such a model exists, remains an open question. As we will discuss, many common players in division mode regulation seem to display specific behaviors that might differ in detail between vertebrate species, context or cell type. As introduced, there is also temporal progression of division modes in the CNS, and the mechanisms underlying this temporal regulation, when known, will also be discussed.

### 4.1 Apical Domain

NECs and RGCs possess a small apical domain that contains the primary cilium, which is nucleated from the mother centriole and protrudes into the brain ventricle to detect signalling molecules [reviewed by (Wilsch-Bräuninger and Huttner, 2021)]. The apical membrane is delineated by polarity and junctional complexes that tether the NPCs to the ventricular surface. These complexes are important to maintain neuroepithelial integrity and normal layering in the cortex [reviewed by (Veeraval et al., 2020)].

Upon cell division, the cleavage furrow is oriented towards the apical domain that is subsequently divided between the daughter cells. Initial studies indicated that asymmetric division of RGCs is accompanied by the cleavage furrow bypassing the apical membrane, dividing the daughter cells in apical domain-inheriting and non-inheriting cells (Figure 2C) (Kosodo et al., 2004). However, careful inspection of live imaging data in several studies has shown that bypassing of the apical membrane is not an absolute property of asymmetric division [(Shitamukai et al., 2011; Fujita et al., 2020)]. Instead, it appears that in asymmetric neurogenic divisions, the apical domain is often equally bisected, and the apical domain and junctions are disassembled later on in the differentiating daughter cell (Figure 2D).

While attachment to the ventricular surface is an important property of apical NPCs, there seem to be a clear distinction between the polarity and the junctional components of adherens junctions (AJs), as polarity proteins seem to have a role in division mode selection that has not been observed when junctional proteins such as N-cadherin are disrupted, despite its clear importance in keeping NPC in the proliferative niche near the ventricle (Miyamoto et al., 2015; Veeraval et al., 2020).

### 4.2 Polarity Proteins

Inheritance of cell cortex factors and polarity proteins is one of the best characterized mechanisms to introduce asymmetry in sister cells. As in all epithelia, NPCs are closely connected to each other through cell-cell contacts. These apical contacts are composed of junctional proteins such as cadherins and catenin and polarity complexes such as Par3-aPKC-Par6. Studies in the developing mouse cortex showed that cortical mPar3 can be symmetrically or asymmetrically inherited in sister cells, independently of cleavage plane orientation (Bultje et al., 2009). In oblique divisions, Par3 can be inherited towards the most apical cell or towards the more basal cell. This association between apical Par3 inheritance and maintenance of proliferative capacity was recently shown to occur in zebrafish forebrain NPCs as well (Zhao et al., 2021). This contrasts with previous reports from asymmetric divisions in the zebrafish spinal cord and the zebrafish hindbrain, where inheritance of apical Par3 was biased towards the neuronal daughter cell (Alexandre et al., 2010; Kressmann et al., 2015). The exact reason underlying these regional differences is unknown.

In the developing mouse cortex, there is a progressive downregulation of cadherin, Par3, Par6 and aPKC, indicating that their reduction is a key step during neurogenesis (Costa et al., 2008). Disruption of Par3 expression leads to an increase in symmetric divisions at the expense of asymmetric divisions. It seems that disruption of Par3 promotes either proliferative or differentiative divisions depending on the context (Costa et al., 2008; Bultje et al., 2009). Furthermore, disruption of Par3 can also lead to randomization of the spindle orientation (Liu et al., 2018). Overexpression of Par3 and Par6 leads to increased clonal size. In this context, Par3 functions in asymmetric stem cell division and acts upstream of Numb and Numb-like in regulating Notch signaling. Research indicates that Par3 can also regulate the activity of the pro-proliferative Hippo pathway in conjunction with Notch signaling (Liu et al., 2018). This shows that asymmetrical inheritance of polarity proteins influences cell fate through its effect on signaling activity, in particular upstream of Notch signaling.

Another unique property of NPCs that is connected to their apicobasal polarity is the presence of a basal extension.

### 4.3 Basal Process

The basal process is an extension of the cell body that connects NPCs to the pial surface through its basal endfoot (Figure 2B). In cortical development, it serves as scaffolding and support for neuronal migration. It is a dynamic structure. In mitosis, the basal process is not disassembled, but rather thins out and acquires a thread-like appearance. Subsequently, it can be split into two and inherited symmetrically by the two daughter cells (Figure 2A), or inherited by only one of the daughter cells (Figures 2B–D). However, even when inherited asymmetrically, in some cases, daughter cells can regrow a basal process after cell division and remain progenitors (Figure 2C; see also Section 5.1). As discussed above, retention of the basal domain is one of the determining factors in maintenance of self-renewing capacity and cortical expansion through increased divisions of basal RGCs (bRGCs) [reviewed by (Kalebic and Huttner, 2020)].

**FIGURE 3:**
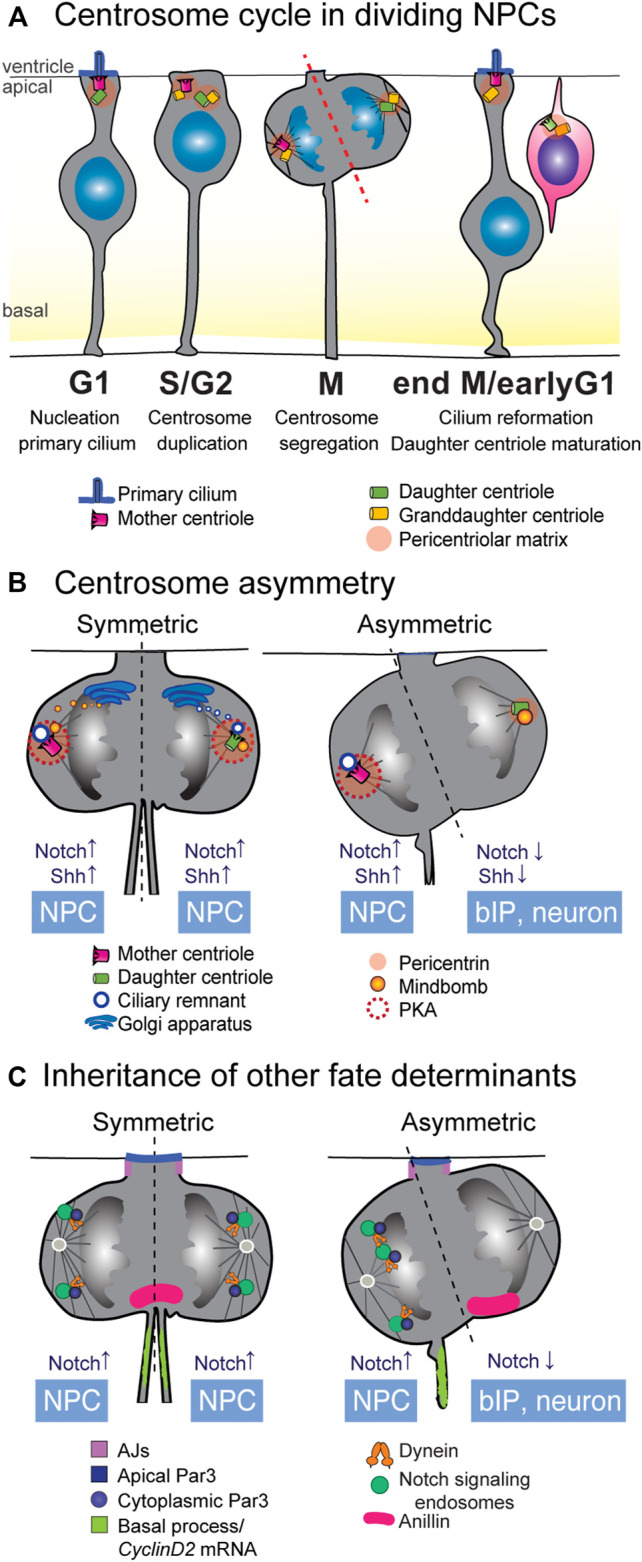
Inheritance of cellular components as fate determinants. **(A)** Centriole duplication cycle in NPCs. In NPCs in interphase and G1, the mother centriole doubles as the basal body for anchoring of the primary cilium. As the cell cycle progresses through S and G2 phases, the centrioles within the centrosome disengage and the centrosome duplicates in a semi-conservative manner. In mitosis, one of the microtubule spindle poles is nucleated by the mother centrosome, containing the fully mature mother centriole, while the opposite spindle emanates from the immature daughter centriole. In P-N divisions, inheritance of the fully mature mother centriole is often correlated with acquisition of a P fate. **(B)** Several aspects of centrosome biology show asymmetry in P-N divisions (left), and there are active mechanisms by which these asymmetries are corrected in P-P divisions. Early P-P divisions show higher levels of pericentriolar matrix, which recruits PKA to the centrosomes and promotes Shh signaling. Inheritance of the ciliary membrane allows cells to quickly regrow a cilium after cytokinesis, promoting a P fate. In early P-P divisions, *de novo* ciliary membrane is proposed to derive from the Golgi and dock to the daughter spindle pole before cell division, equalizing the speed of cilium reformation. Notch modulator Mindbomb1 associates with the daughter centriole and its inheritance correlates with N fate in P-N divisions. In early P-P divisions, a pool of Mindbomb1 is released from the Golgi towards the mother centrosome spindle. **(C)** Many cellular components show specific patterns of behavior in P-P and P-N divisions. Inheritance of the basal process is known to be correlated with P fate, at least partly due to the presence of *CyclinD2* mRNA that can facilitate cell cycle re-entry. The apical polarity protein Par3 can be asymmetrically inherited in mitosis, albeit its correlation with specific fates is context-dependent. Inheritance of the midbody protein Anillin correlates with N fate. Asymmetric distribution of endosomes containing Notch signaling molecules occurs in mitosis and is dependent on dynein and Par3. It is suggested that differences in the abundance of spindle microtubules can underlie this asymmetric distribution. bIP, basal intermediate progenitor; NPC, neural progenitor cell.

The basal process does not only have an architectural role, but it also serves in fate determination. In asymmetric divisions, the cell inheriting the basal process retains the stemness character. Research has shown that mRNAs can be specifically transported to the basal process (Pilaz et al., 2016; Tsunekawa et al., 2012). For instance, mRNA for the cell cycle factor *CyclinD2* is specifically localized in the basal process, and daughter cells that inherit the basal process containing *CyclinD2* mRNA remained progenitors more often than not, showing that this promotes self-renewing capacity (Tsunekawa et al., 2012) (Figure 3C). The RNA-binding protein FMRP was recently shown to transport several mRNAs to the basal endfoot, which subsequently are locally translated (Pilaz et al., 2016). This suggests that the basal endfoot might be locally regulated through translation and interactions with the basal lamina and neighboring cells (Pilaz and Silver, 2017). It was also proposed that through the basal process, basal signals can be relayed to the NPC’s soma. Indeed, maintenance of the basal process seems to be important for maintenance of proliferative potential, which is exemplified by the fact that the basally located bRGCs that retain some self-renewing capacity possesses a basal process, but no apical attachment (reviewed by Kalebic and Huttner, 2020).

Next to specific inheritance of apical and basal domains, the apicobasal polarity of NPCs is also connected to division mode regulation through changes in the orientation of the mitotic spindle and cleavage furrow.

### 4.4 Spindle Orientation

As briefly introduced above, most vertebrate NPCs divide at the apical surface with a vertical spindle orientation and their cleavage plane perpendicular to the apical surface (Figures 2A,C,D). Spindle orientation and the plane of division change throughout neurogenesis [reviewed by (Pietro et al., 2016)]. In early apical divisions, cells divide perpendicularly to the ventricular surface, thus parallel to the apicobasal axis (Figure 2A). In later neurogenic stages, oblique division planes become more frequent, albeit still in the minority [Figure 2B (Shitamukai et al., 2011)]. This temporal regulation is reminiscent of the division plane shift observed in *Drosophila* neuroblasts, where regulation of the mitotic plane of division is the central mechanism allowing for a switch between symmetric proliferative and asymmetric divisions (Pietro et al., 2016). Moreover, the molecular players regulating spindle orientation are conserved between *Drosophila* and vertebrates. However, in vertebrate development, the orientation of the spindle plane is not the main determinant of the symmetry of a NPC division, as asymmetric fates can and do arise from vertical divisions (Peyre et al., 2011).

The orientation of the mitotic spindle and cleavage plane is regulated by interactions between motor proteins, astral microtubules and the cell cortex. Astral microtubules interact with the cell cortex through microtubule-capture mediated by the LGN/NuMA (protein) complex. In mid-neurogenic stages in the developing mouse neocortex, expression of Inscuteable (Insc) leads to horizontal cleavage planes. Induction of spindle randomization through loss of LGN or overexpression of Insc is followed by increased neuron production and displacement of apical RGCs to a basal position in chick spinal cord (Das and Storey, 2012). This shows that spindle orientation regulation acts in positioning the NPCs within the neuroepithelium (Section 5.3).

Spindle orientation in metaphase is continually changing, and it is not stabilized until anaphase (Peyre et al., 2011; Roszko et al., 2006). Interestingly, the amplitude of variation is higher in neurogenic divisions than in proliferative NPC divisions, which might be at least partially explained by the downregulation of apical polarity and AJ proteins (Figures 2A,D). A specific population of astral microtubules—those reaching the apical and basal cell cortex—decreases in abundance in neurogenic progenitors when compared to symmetric proliferating progenitors (Mora-Bermúdez et al., 2014; Da Silva et al., 2021). The abundance of astral microtubules to reach the cell cortex is regulated by LGN (Mora-Bermúdez et al., 2014). In NPCs, the tight junction protein Occludin interacts with NuMA, indicating coupling between the junctional belt and the cortical machinery. Mice lacking a long isoform of Occludin show fewer astral microtubules, increased genomic instability and apoptosis (Bendriem et al., 2019), which authors suggest might be due to an elongation of the M-phase which is known to induce premature cell cycle exit and differentiation. Thus, defects in regulation of the cleavage plane can lead to an increase in neurogenesis through lengthening of the cell cycle as a side effect of mitotic challenges, rather than a direct effect on the mechanism of division modes.

Interestingly, many known mutations linked to microcephaly (smaller brains) in humans are related to centrosomal genes, which are implicated in spindle orientation regulation particularly in early symmetric expanding divisions (Marthiens and Basto, 2020). Moreover, mutations affecting spindle orientation can lead to an increase in asymmetric divisions (Konno et al., 2008; Fujita et al., 2020), possibly by inducing the asymmetric inheritance of fate determinants such as the apical membrane or the basal process. However, segregation of the apical membrane also seems to be independent from the cleavage plane (Kosodo et al., 2004), as, e.g., apical polarity components can be asymmetrically inherited even in perpendicular divisions, indicating that there are additional mechanisms regulating their inheritance.

### 4.5 Centrosome Asymmetry

The centrosome is the main microtubule organizing center of animal cells, and as such it has crucial functions in spindle formation, vesicle transport and cell signaling through the primary cilium. In agreement with its variety of cellular functions, the centrosome plays key roles in NPC biology. An indication of the central role of centrosomes in brain development is that many of the genes mutated in neurodevelopmental disorders such as microcephaly are centrosomal genes (reviewed by Marthiens and Basto, 2020). Some of those mutations cause microcephaly by affecting spindle pole orientation, leading to premature depletion of the progenitor pool as has been previously discussed. Some cause mitotic abnormalities which in turn activate progenitor cell apoptosis. The depletion of centrioles in a p53-null background leads to loss of the apical attachments and displacement of progenitors to a basal location in the mouse developing cortex (Insolera et al., 2014). Presence of supernumerary centrioles in the developing mouse neocortex and zebrafish developing brain leads to microcephaly due to increased multipolar spindles and apoptosis of NPCs (Marthiens et al., 2013; Hu et al., 2014; Dzafic et al., 2015). Together this suggests that regulation of centriole number and functioning is important in maintenance of NPCs.

In the G_1_-phase of the cell cycle, the centrosome is anchored at the ventricular surface where it serves as the basal body for the nucleation of a primary cilium that extends towards the signaling-rich environment of the ventricular lumen [Figure 3A (Wilsch-Bräuninger and Huttner, 2021)]. In G_2,_ the cilium retracts and centrioles duplicate in a semi-conservative manner: the former basal body templates a new centriole and becomes the mother centrosome, while the daughter centriole and the newly formed granddaughter centriole form the daughter centrosome [Figure 3A; see a recent review (Blanco-Ameijeiras et al., 2022)]. Notably, the mother and daughter centrosomes are not structurally and functionally equivalent, as the mother centrosome has already undergone the gradual process of centriole maturation and is decorated with appendage proteins important for nucleation of the primary cilium and for microtubule nucleation (Kumar and Reiter, 2021). The daughter centrosome will not fully mature until one and a half cell cycles later (Figure 3A). Each centrosome nucleates one half of the spindle pole and is segregated into the sister cell in mitosis. After cytokinesis, daughter cells can regrow a primary cilium, with the mother centriole-inheriting cells establishing a cilium that can respond to Shh signaling before its sister cell (Anderson and Stearns, 2009; Piotrowska-Nitsche and Caspary, 2012). In this way, the centriole duplication cycle leads to an asynchrony in centriole age, and thus, a functional asymmetry in maturation state between old mother centrioles and newly maturing (ex-daughter) centrioles (Figures 3A,B).

The inherent functional asymmetry of the mother and daughter centrosomes can be co-opted to introduce cellular asymmetries in daughter cells that can ultimately translate into fate asymmetries [reviewed by (Saade et al., 2018; Wilsch-Bräuninger and Huttner, 2021)]. This was first observed in the asymmetric division of *Drosophila* male germline cells, which divide in such a way that one of the daughter cells remains attached to the hub cells, becoming a new germline stem cell, whereas the other cell is born outside of the niche and becomes a gonioblast [reviewed by (Venkei and Yamashita, 2018)]. In these cells, the mother centriole is retained always in the new germline stem cell, purportedly due to the higher microtubule nucleation capacity of the mother centriole connecting it to the AJs. A stereotypical pattern of centrosome inheritance has since also been shown in the mouse neocortex, where the mother centriole is preferentially inherited by the daughter cell that will retain the progenitor potential, whereas the daughter centriole is inherited by the newborn neuron [Figure 3A (Wang et al., 2009; Paridaen et al., 2013)]. It is important to note that not in all cases of asymmetric stem cell division, the stem cell inherits the mother centriole. A good example are *Drosophila* neuroblasts, where the daughter centrosome is inherited by the prospective neuroblast. In the dividing cell, the mother centriole quickly loses MTOC functionality and the daughter centriole quickly acquires it. In vertebrates, random inheritance of centrioles was observed in cerebellum granule neuron precursors (Chatterjee et al., 2018). Together, these findings show an evolutionary conserved correlation between stereotypical centrosome inheritance, asymmetric cell division and cell fate specification in the developing brain.

Subsequent studies have shed light on what particular characteristics of the mother centriole might contribute to promoting stemness in vertebrates. For one, studies have shown that mutations in mother centriole proteins can lead to failure to maintain progenitor ability and induce premature differentiation, probably by promoting detachment from the apical surface (Jayaraman et al., 2016). Importantly, recent studies have demonstrated that mutations of the mother centriole protein Cep83 leads to macrocephaly and expansion of the progenitor pool by increasing apical membrane stiffness and activating the Hippo signaling pathway component YAP that promotes proliferation (Shao et al., 2020). Therefore, it appears that next to cilium nucleation and MTOC functioning, the mother centriole also acts in ensuring adequate apical attachment through controlling mechanical properties of the apical domain.

An additional mechanism whereby the mother centriole favors a progenitor fate is by retaining a fragment of the internalized ciliary membrane throughout mitosis (Figure 3B) (Paridaen et al., 2013). Cells that inherit the ciliary membrane remnant are faster in re-growing a cilium after division than its sister cell, and thus show active ciliary-mediated Shh signaling earlier than the sibling cell. Ciliary membrane inheritance is furthermore associated with retention of the stem cell character in asymmetric cell divisions. An interesting observation here is that the mother centriole and basal process are preferentially co-inherited during mitosis (Figure 3B), which might suggest an intracellular connection between the two through the cytoskeleton. Asymmetric ciliary remnant inheritance appears to be conserved in vertebrate neurogenesis as it has also been observed in the chick spinal cord (Saade et al., 2017). In the mouse neocortex, in cells destined to remain NPCs, the cilium reforms at the apical membrane, whereas in differentiating neurons, the cilium is established on the basolateral side (Wilsch-Bräuninger et al., 2012). These findings suggests that spatiotemporally controlled asymmetric ciliogenesis coupled to asynchronous Shh signaling is an evolutionary conserved mechanism in NPC divisions.

Taken together these finding, centrosome asymmetries have been found to determine NPC division mode and daughter cell fates through mechanisms that affect positioning within the tissue and synchrony of signaling states (Figure 3B). To which extent these mechanisms co-exist in all individual NPCs population or whether they are region-specific is still unclear. Moreover, centriole asymmetries might also be connected to asymmetries in other fate determinants, such as recycled signaling components. Recently, temporal changes to overcome centriole age asymmetry have been identified that are connected to signaling activities [reviewed by (Gonzalez, 2021)], which we will discuss in Section 5.2.

### 4.6 Endosomes

Endocytosis and recycling of ligands and receptors at the plasma membrane is a rapid and flexible mechanism to modulate cell signaling. Asymmetric distribution of signaling endosomes during mitosis can impose signaling asymmetries in sister cells even before cytokinesis is fully complete [reviewed by (Daeden and Gonzalez-Gaitan, 2018)]. One the best characterized examples of endocytosis regulating symmetry is the regulation of Delta-Notch signaling in sensory organ precursors (SOP) cells in *Drosophila*, where a SOP undergoes an asymmetric division that produces two different precursors cells: pIIa (Notch ON) an pIIb (Notch OFF), that will go on to produce different types of cells (Daeden and Gonzalez-Gaitan, 2018). Establishment of this asymmetry in Notch signaling is determined by several concomitant mechanisms, and among those, endosomal compartments that harbour the ligand Delta and the receptor Notch feature prominently. In mitosis, Rab11+ recycling endosomes distribute symmetrically, but after cytokinesis, they accumulate around the centrosome in the pIIb cell, promoting recycling of Delta in pIIb and subsequent Notch activation in pIIa (Emery et al., 2005). Additionally, Sara endosomes—early endosomes that contain Notch and Delta and in which active Notch signaling can take place—are unequally distributed in cytokinesis and biased towards the pIIa cell (Coumailleau et al., 2009). Asymmetric distribution of Sara endosomes is reported to be caused by asymmetric microtubule density which directs more Sara endosomes towards the pIIa cell (Derivery et al., 2015).

Asymmetric dispatch of Sara endosomes has been shown in other asymmetric stem cell division systems, including asymmetric divisions of neural progenitors in the zebrafish spinal cord (Kressmann et al., 2015; Montagne and Gonzalez-Gaitan, 2014). In that study, authors investigated the partition of Sara endosomes in mitosis and found that the cell inheriting a higher amount of Sara endosomes was most of the time destined to become the progenitor cell [Figure 3C, right panel (Kressmann et al., 2015)]. It is important to note that asymmetry in Sara endosome segregation was not predictive of the symmetry or asymmetry of a division, as asymmetric Sara endosome dispatch occurred in symmetric proliferative divisions and asymmetric P-N divisions occurred even with low levels of Sara endosome asymmetry. Moreover, Sara mutants showed no difference in the number of P-P divisions, but an increase in differentiative N-N divisions at the expense of asymmetric P-N divisions, indicating that in P-N divisions, Sara is important for the acquisition of a progenitor fate. This contrasts with a recent study in Sara endosomes in asymmetric neural progenitor divisions in the zebrafish retina, where progenitors divide to produce an uncommitted progenitor (Notch High) and a neurogenic progenitor (Notch Low) (Nerli et al., 2020). Similarly to the spinal cord, Sara endosomes show asymmetric dispatch and its inheritance correlates with high Notch activity and pluripotency (Figure 3C). However, disruption of Sara led to an increase in Notch activity and in symmetric proliferative divisions. This indicates that most likely, the precise way in which Sara endosomes modulate Notch signaling is context-dependent, as several other possibly competing mechanisms influence Notch signaling simultaneously.

Other mechanisms in asymmetric cell division that we have mentioned earlier are also connected to Sara endosomes. In the spinal cord, Sara endosomes and apical Par3 segregate to opposite cells, partially corroborating previous results that apical Par3 correlates with neuronal fate in asymmetric P-N divisions (Kressmann et al., 2015). Moreover, the E3 ubiquitin ligase Mib1 that is required for Delta endocytosis, and DeltaD both were found associated with Sara endosomes, suggesting a cell-autonomous Notch activation. This appears to be different in the zebrafish forebrain: a recent study looked at endosomes containing internalized DeltaD and found them to segregate to the Notch-high cell independently and opposite to Mib1 (Zhao et al., 2021). Notably, here, apical Par3 also segregates to the Notch High cell. This study also offered some key insight into how asymmetric partition of internalized DeltaD endosomes is achieved, and found that similarly to Sara endosomes, internalized DeltaD endosomes localized at the center of the spindle in anaphase show asymmetric distribution in telophase. This asymmetric distribution is mediated by the dynein motor complex, and the authors found that a cytosolic pool of Par3 previously thought to be inert, is in fact responsible for engaging dynein in endosomal transport (Figure 3C). It is not known at the moment whether the asymmetry exists at the level of the Par3 cytosolic pool or, similarly to fly SOPs (Daeden and Gonzalez-Gaitan, 2018), at the level of microtubule density, leading to biased trafficking towards one pole.

Together, these studies indicate that intracellular asymmetries in distribution of endosomes containing Notch signaling components plays an important role in determining division outcomes (Figure 3C). Interestingly, the exact connection between inheritance of endosomes harboring different Notch components is not absolute and seems to be context-dependent, which could also be related to Notch signaling events occurring in *cis* (within a cell) versus in *trans* between neighboring cells (Baek et al., 2018; Nerli et al., 2020).

### 4.7 Midbody

In dividing NPCs, the cytokinetic furrow ingresses from the basal side toward the apical side, ending with the partition of apical membrane components that we discussed already. The midbody, a temporary structure that is formed in cytokinesis when the actomyosin cytoskeleton constricts around the microtubule bridge, can persist after cytokinesis and can be symmetrically or asymmetrically inherited [reviewed by (Dionne et al., 2015)]. Recent studies have suggested that midbodies can be internalised and can act as an intracellular signaling platform (Peterman et al., 2019). Interestingly, in cultured cells, midbodies were preferentially inherited by the daughter cell that also inherits the mother centriole (Kuo et al., 2011), suggesting that the cytoskeleton asymmetries connected to centriole age asymmetry might play a role in specific midbody inheritance.

In NPCs in mammalian cortical development, there is a bilateral abscission of the midbody remnant. Midbody remnants are much more abundant in early cortical progenitors than in late stage cortical progenitors, and maintenance of the midbody remnant is slightly correlated with symmetric proliferative divisions (McNeely and Dwyer, 2020). In the zebrafish retina, Anillin, an F-actin binding protein with important roles in the midbody, is inherited asymmetrically in 60% of divisions (Paolini et al., 2015). In those divisions, the cell inheriting Anillin retracts its apical process and migrates basally, and asymmetric distribution of the apical protein Par3 is dependent on Anillin (Figure 3C). Hypomorphic Anillin mutants showed an increase in symmetric neurogenic division at the expense of proliferative divisions, and at the transcriptional level, the retinal neuronal marker Atoh5 downregulates Anillin.

Together, this suggests that midbodies indeed play a role in NPC proliferation, though more studies are necessary to elucidate the underlying mechanisms further.

### 4.8 Other Fate-Determining Factors

Taken together, the research results summarized above provide an overview of the currently known NPC biological features that play a role in division mode selection. In other stem cell systems, additional fate-determining factors have been identified that are asymmetrically segregated between daughter cells [see a recent review by (Sunchu and Cabernard, 2020)]. For example, specific segregation of old mitochondria to the differentiating daughter cells was observed in human mammary epithelial cells (Katajisto et al., 2015). Other cellular asymmetries that have been associated with division outcomes in other stem cell types such as *Drosophila* intestinal and germ stem cells are specific segregation of histones and sister chromatids (Ranjan et al., 2019; Wooten et al., 2020). Whether asymmetric segregations of these organelles and structures also plays a role in NSCs in vertebrates is currently unclear or controversial.

What is clear from recent findings is that asymmetric segregation of organelles and molecules is often interconnected. For example, the polarity protein Pard3 that localizes mainly near AJs also is localized near Sara endosomes and plays a role in their intracellular transport, which influences Notch signaling asymmetries (Zhao et al., 2021). Therefore, an intriguing open question is whether and how the different intracellular fate-determining factors and structures interact and depend on each other. If such fate determining factors act independently, what is the weight of each of them towards division mode selection? As we have discussed before, asymmetric segregation of fate determinants could be expected to act deterministically in selection of division mode. However, in reality, the occurrence of asymmetric segregation of fate determinants is not absolute. For instance, asymmetric retention and inheritance of the primary cilium remnant occurs in about 70%–80% of all mouse neocortical NPCs in early neurogenic stages (Paridaen et al., 2013; Jayaraman et al., 2016). This raises the exiting possibility that NPC subtypes [(Fischer and Morin, 2021; Ortiz-Álvarez and Spassky, 2021), see also Section 3] using different combinations of fate-determinant inheritance mechanisms might exist. Another explanation is that these mechanisms are influenced by stochastic processes, which could lead to higher heterogeneity in their prevalence in a population of more or less equipotent and similar NPCs. Future experiments will hopefully shed more insight into these open issues.

## 5 What Determines Whether a NPC Division is Symmetric or Asymmetric?

Since individual neural progenitors use both symmetric and asymmetric division modes in their lineages, an obvious question is how the symmetry of the division is determined, and how inherent asymmetries connected to asymmetric division are overcome to allow symmetric division outcomes. In relatively more expanded areas of the brain such as the mammalian neocortex, a higher neuronal production per individual NSC is ensured through spindle orientation changes that underlie generation of specialized basally positioned intermediate progenitors (Uzquiano et al., 2018; Llinares-Benadero and Borrell, 2019; Kalebic and Huttner, 2020). Recent work has provided new insights into how progenitor properties change over time to ensure the proper balance of self-renewing and intermediate progenitors, and differentiating cells as the brain grows.

### 5.1 Switch From Pre-Neurogenic Symmetric Division to Neurogenic Asymmetric Divisions

The first obvious change in NPC division modes takes place when pre-neurogenic neuroepithelial cells (NECs) switch from their initial symmetric proliferative divisions (Figure 2A) to asymmetric neurogenic divisions (Figures 2B–D). During this switch, morphological and molecular changes in cell-cell junctions, cell shape and onset of glial cell markers occur (Taverna et al., 2014; Uzquiano et al., 2018). After this transition, these progenitors are commonly named radial glial cells (RGCs). NECs are wide and columnar in shape, whereas RGCs are more slender and elongated. Recent work showed that in primates, the NEC-to-RGC-transition is gradual and involves subtle cell shape changes induced by apical constriction and changes in polarity that is mediated by transient expression of the epithelial-mesenchymal transition (EMT) factor *ZEB2* during the transition, which induces apical constriction through regulation of the actin-regulator Shroom3 (Benito-Kwiecinski et al., 2021). Similarly, in the zebrafish embryonic retina, expansion of the apical domain surface by inhibition of Shroom3 or loss of Llgl1 led to increased Notch activity and diminished neurogenesis (Clark et al., 2012). This suggests that regulation of the apical domain through apical constriction can influence Notch signaling activity, which in turn affects cell fate. However, it is currently still unclear whether and how NEC to RGC transition morphological changes are directly coupled to onset of asymmetric divisions.

As mentioned already, several morphogens and signaling molecules play a role in regulation of NEC self-renewal and the onset of neurogenesis [reviewed by (Agirman et al., 2017)]. Some of these signaling molecules are expressed or secreted at specific locations within the developing brain, leading to anteroposterior and dorsoventral gradients of signaling activity. For instance, in the mammalian forebrain, the anterior neural ridge secretes several Fibroblast Growth Factors (Fgfs) that influence NSC proliferation and division mode. For instance, Fgf2 shortens the cell cycle and promotes symmetric divisions *in vitro* (Ledesma-Terrón et al., 2020). Fgf10 is transiently expressed by cortical progenitors during NEC to RGC transition. Depletion of Fgf10 extends the pre-neurogenic symmetric proliferative period and delays expression of RGC markers and neurogenesis (Sahara and O’Leary, 2009). Delta-Notch signaling is involved in initiation of neurogenesis. Through salt-and-pepper patterns of proneural gene and Delta ligand expression, Notch signaling is activated in neural progenitors, which supports their proliferation [reviewed by (Kageyama et al., 2019)]. Subsequently, through lateral inhibition between neighboring cells and sister cells, asymmetric neuronal daughter fate is established [reviewed by (Moore and Alexandre, 2020]. Thus, timely onset of Fgf and Notch signaling are key to generating neurogenic radial glial cells that are able to undergo asymmetric divisions. At the same time, the NEC to RGC transition is gradual and in early neurogenic stages, symmetric divisions do still occur, albeit with lower prevalence (Gao et al., 2014).

Even though asymmetric segregation of polarized cell structures is an important mechanism to mediate asymmetric fates in early neurogenic stages, early RGCs appear to have some (latent) capacity to overcome these asymmetries. In mice and human samples, it was shown that early neurogenic stage-RGCs possess the capacity to regrow an apical process in divisions where the apical domain was inherited by the differentiating cells [Figure 2C, (Fujita et al., 2020; Shitamukai et al., 2011; Subramanian et al., 2017)]. This ability is dependent on Notch/Integrin-beta1 pathways and is linked to high levels of vesicle transport and recycling of junctional proteins and membranes in early-stage RGCs (Fujita et al., 2020). Similarly, splitting or regrowth of the basal process in the non-inheriting cell has been observed specifically in early neurogenic stages (Kosodo et al., 2008; Shitamukai et al., 2011). Similar to regrowth of the apical domain, re-establishment of the basal process is increased upon forced activation of Notch signaling [Figure 2C (Shitamukai et al., 2011; Fujita et al., 2020)]. In conclusion, at early stages when symmetric division predominates, NPCs are able to generate daughter cells that eventually are more symmetric in terms of apical and basal domains (through inheritance and fast re-growth) than their initial division asymmetry. NPCs appear to lose these abilities as embryonic neurogenesis proceeds towards mid- and late neurogenic stages.

Taken together, gradual changes in the morphology and ability to re-establish apical and basal domains appear to underlie the reduction of symmetric proliferative division modes as neurogenesis proceeds. A next question is how symmetric division modes can be reconciled with intrinsic cellular asymmetries.

### 5.2 Inherent Cellular Asymmetries and Symmetric Division Outcomes

Dynamic control of the asymmetric inheritance of cellular components in mitosis appears to be a fairly straightforward way to establish asymmetric daughter cell fates. The question arises however how inherent cellular asymmetries such as centriole age differences, are overcome in symmetric divisions. In theory, the centriole asymmetry could be compensated for by making centrioles more equal. This could be done in several ways. For instance, specific proteins and thereby functions of the mother centriole could be removed prior to or during mitosis [as observed in *Drosophila* neuroblasts (Marthiens and Basto, 2020; Gonzalez, 2021)] or maturation of the daughter centriole into a new mother centriole could be sped up (Blanco-Ameijeiras et al., 2022). Furthermore, regulation of the pericentriolar matrix (PCM) composition, which couples the centriole to the microtubule network, could also influence centriole symmetry (Kumar and Reiter, 2021). Such measures would enable functional symmetry through regulation of more synchronous primary cilium reformation, symmetric signaling states, and microtubule nucleation and anchoring, and transport of vesicles.

Recent studies have provided evidence that these mechanisms generating centriole symmetry indeed play a role in early pre-neurogenic symmetric divisions and that this involves signaling cascades [see also (Gonzalez, 2021) (Figure 3B)]. For example, Notch signaling components have been shown to associate specifically with centrosomes. In the chick neural tube, Mib1 that is inherited asymmetrically by the differentiating daughter cell, was found to associate specifically with the daughter centriole through interaction with the centriolar satellite proteins PCM1 and Azi1 (Tozer et al., 2017). In symmetric proliferative divisions, a pool of Mib1 emanating from the Golgi apparatus docks to the mother centrosome during division, leading to more symmetrical Notch signaling between sister cells (Figure 3B). Involvement of Golgi-derived trafficking in overcoming centriole asymmetries was also suggested during pre-neurogenic stages of mouse neocortex development. Here, higher occurrence of symmetrically localized primary cilium components as observed at both centrosomes, one of which presumably constitutes recycled ciliary membrane and the other *de novo* synthesized ciliary membrane (Paridaen et al., 2013) (Figure 3B). Together, this suggests that delivery of Golgi-derived vesicles can compensate for centriole asymmetry during symmetric divisions.

One remaining question is how these temporal centrosomal and Golgi dynamics are regulated at the transcriptional level. Another study in the chick spinal cord offers some clues on the transcriptional regulation underlying centrosome functional asymmetry. In chick spinal cord and mouse cerebellum, high levels of Shh signaling maintain symmetric proliferative divisions [Figure 3B (Merk et al., 2020; Saade et al., 2013)]. In the chick spinal cord, overactivation of Shh increases symmetric proliferative divisions at the expense of symmetric neurogenic divisions (Saade et al., 2013). A recent follow-up study showed that high Shh signaling activity in NECs increases pericentrin levels at both centrosomes (Figure 3B, left panel). This mediates symmetric sequestering of PKA at both centrosomes (Saade et al., 2017), which in turn enhances Shh signaling to promote symmetric proliferative divisions. In contrast, at later stages when asymmetric divisions occur, PKA distribution at centrosomes was unequal (Figure 3B, right panel). Crosstalk of Notch and Shh signaling was also observed in primary cilia that are nucleated by the mother centriole. In the mouse spinal cord, Notch signaling restricts localization of the Shh receptor Patched and promotes accumulation of the Shh component Smo in primary cilia (Kong et al., 2015). In this way, Notch signaling controls the response of NPCs to Shh signals. Together, these findings suggest that signaling activities can induce differential recruitment of PCM proteins in order to compensate for centriole asymmetry to ensure symmetry of Shh and Notch signaling pathways in both daughter cells.

Whereas these findings suggest that mechanisms promoting daughter centriole function act in pre-neurogenic symmetric proliferative divisions, at later neurogenic stages, symmetric differentiative divisions occur in which both daughter cells initiate neuronal fate. Here, the question remains as to how centriole functional asymmetries are overcome in generating symmetric daughter cells. Intriguingly, loss of centrosomal-associated ciliary components is associated with later neurogenesis stages. In the mouse neocortex and chick spinal cord, the association of recycled ciliary membrane in mitotic progenitors is often lost, leading to slower and synchronous cilium reformation between sister cells (Paridaen et al., 2013; Saade et al., 2017). Furthermore, apical constriction through apical microtubule rearrangements and abscission of apical domain containing the ciliary membrane occurs prior to delamination of nascent neurons in chick spinal cord (Das and Storey, 2014; Kasioulis et al., 2017), suggesting that loss or later establishment of a functional primary cilium is important for delamination and differentiation of neurons. Microtubule re-organisation and apical constriction similar to epithelial-to-mesenchymal transition (EMT) through action of the mother-centriole specific protein Akna also plays a role in delamination of intermediate progenitors in the mammalian cortex (Camargo Ortega et al., 2019). This indicates that regulation of the attachments between mother centriole and primary cilium play a key role in neuronal cell fate specification.

Taken together, recent studies have provided some insights into how signaling activity can differentially affect the functional outcome of centriole age asymmetry through intracellular trafficking of centrosome satellite proteins. It would be interesting to learn more about how signaling activity levels are connected to processes such as fate determinant inheritance, intracellular trafficking, centriole composition and nucleation of primary cilia and microtubules by the mother centrioles at different developmental stages. An important open question in this context is what is the role of the timing of signaling activity relative to the cell cycle in determining individual NPC division modes. Though there is little information on this available, a study in chick spinal cord suggested that Notch activity prior to mitosis is connected to immediate activation of Notch activity in both daughter cells, whereas divisions with no prior active Notch signaling were linked to asynchronous and asymmetric Notch activity states between the daughter cells (Vilas-Boas et al., 2011). Another open question is whether localized signaling events can influence the orientation of the mitotic spindle through centriole age asymmetry, as has been demonstrated for Wnt3a signals in cultured embryonic stem cells (Habib et al., 2013).

### 5.3 Spatiotemporal Regulation of Spindle Orientation and NPC Diversity

As discussed earlier, regulation of spindle orientation is an important mechanism in division asymmetry (Figures 1B, 2B). Interestingly, properties of the mitotic spindle and astral microtubules have been found to change as neurogenesis proceeds. For example, astral microtubules are more plentiful in early symmetric divisions and decrease in neurogenic RGCs (Mora-Bermúdez et al., 2014). This mechanism is proposed to restrict wobbling of the mitotic spindle, and thus, the chance of division asymmetry. In contrast, the density of spindle microtubules of neocortical RGCs increases from early to late-neurogenesis (Vargas-Hurtado et al., 2019). Loading of the microtubule nucleation factor Tpx2 to the spindle MT increases over time and appears to be linked to a higher fidelity of chromosome segregation during mitosis at later neurogenic stages. Asymmetries in spindle size within dividing NPCs has also been observed in mouse developing neocortex (Figure 3C). Here, the planar cell polarity regulators Wnt7a and Vangl2 promote asymmetric spindle size that peaks at mid-neurogenic stages. The larger spindle size is associated with neuronal cell fate and the smaller with RGC fate (Delaunay et al., 2014). Together, these findings indicate that astral and spindle microtubule properties are important determinants in restricting spindle orientation deviations and susceptibility to chromosome mis segregation, particularly in early neurogenic stages.

When the spindle orientation is randomized through experimental manipulations, in general this leads to an increase in NPCs that localize more basally away from the ventricle. For example, randomization of the mitotic spindle orientation can be induced through overexpression of Insc or depletion of LGN. Interestingly, the effect of such manipulations show regional and stage-dependent differences. Experiments in the mouse ventral and dorsal telencephalon using acute Insc or LGN manipulations show that these manipulations only affect the spindle of RGCs during mid- and late neurogenic stages, and not in early neurogenic, postnatal and adult stages (Falk et al., 2017). In the mouse ganglionic eminence (the ventral telencephalon), spindle randomization is linked to increased symmetric divisions generating another type of progenitor that lack basal processes, called apical intermediate (aIPs) or short neural progenitors (Falk et al., 2017). These aIPs have apical domains and remain anchored at the ventricular surface, but are not able to re-establish basal processes and typically only generate neurons (Falk et al., 2017; Ramos et al., 2020). These aIPs are also present in the dorsal telencephalon, where their generation depends on temporary expression of the non-canonical tubulin Tuba8 downstream of Fgf10 signaling (Ramos et al., 2020). In the dorsal telencephalon, spindle orientation change is key to the production of neocortical bRGCs in a classis type of asymmetric RGC division (Figure 2B). bRGCs are born from horizontal divisions that start occurring at mid-neurogenic stages in the mammalian neocortex [reviewed by (Kawaguchi, 2021)]. The cleavage plane dissects the dividing cell into one daughter with apical constituents and one daughter cell inheriting the basal process which is important for their self-renewing capacity [Figure 2B (Shitamukai et al., 2011; LaMonica et al., 2013)].

A recent study showed that the time window restriction for bRGC generation to occur only from mid-neurogenic stages is due to a higher capacity of early aRGCs in the mouse neocortex to re-establish an apical domain upon spindle randomization [Figure 2C (Fujita et al., 2020)]. In this way, at early neurogenic stages, daughter cells without apical domains are able to re-establish their incorporation into the ventricular zone junctional belt. Taken together, these findings show that division asymmetry induced by spindle orientation (Figure 2B) coupled to the differential abilities of neural progenitors to re-establish apical or basal domains (Figure 2C) is an important factor in establishing the large diversity of morphological NPC subtypes in the developing mammalian brain.

### 5.4 Regulation of Cell Division in Basal Progenitor Cells

As mentioned in the introduction, basal progenitors (BPs) are the progeny of apical NPCs, and act as amplifying neurogenic progenitors. This strategy of indirect neurogenesis results in an increased neuronal output, and comparative studies in recent years have shown that BPs are in large part responsible for the increased relative neocortical size in humans compared to other primates [reviewed by (Penisson et al., 2019; Kalebic and Huttner, 2020)]. Comparative studies have proved to be especially useful in studying BPs. Here, small interspecies differences in BP biology underlie extremely important evolutionary changes, such as the extent of neocortical folding.

The shared biological feature of these BPs is their ability to divide away from the ventricular surface. Instead, BPs populate the outer sub-ventricular zone (OSVZ), where they establish a progenitor niche that self-expands and generates neurons. Notably, OSVZ relative thickness is much bigger in the neocortices of gyrencephalic species like the ferret than in those of lissencephalic species like mice (Martínez-Cerdeño et al., 2012). In the chicken embryonic dorsal pallium, the region analogous to the mammalian neocortex, a specific domain where non-apical mitosis are abundant has been described (Cárdenas et al., 2018). Interestingly, a very small number of non-apical progenitors have been identified even in the zebrafish embryonic brain and spinal cord (McIntosh et al., 2017). These progenitors share characteristics with mammalian BPs, such as loss of apical attachment and mitosis away from the ventricular surface. Whether these non-apical progenitors are evolutionarily related to mammalian BPs needs further investigation.

**FIGURE 4:**
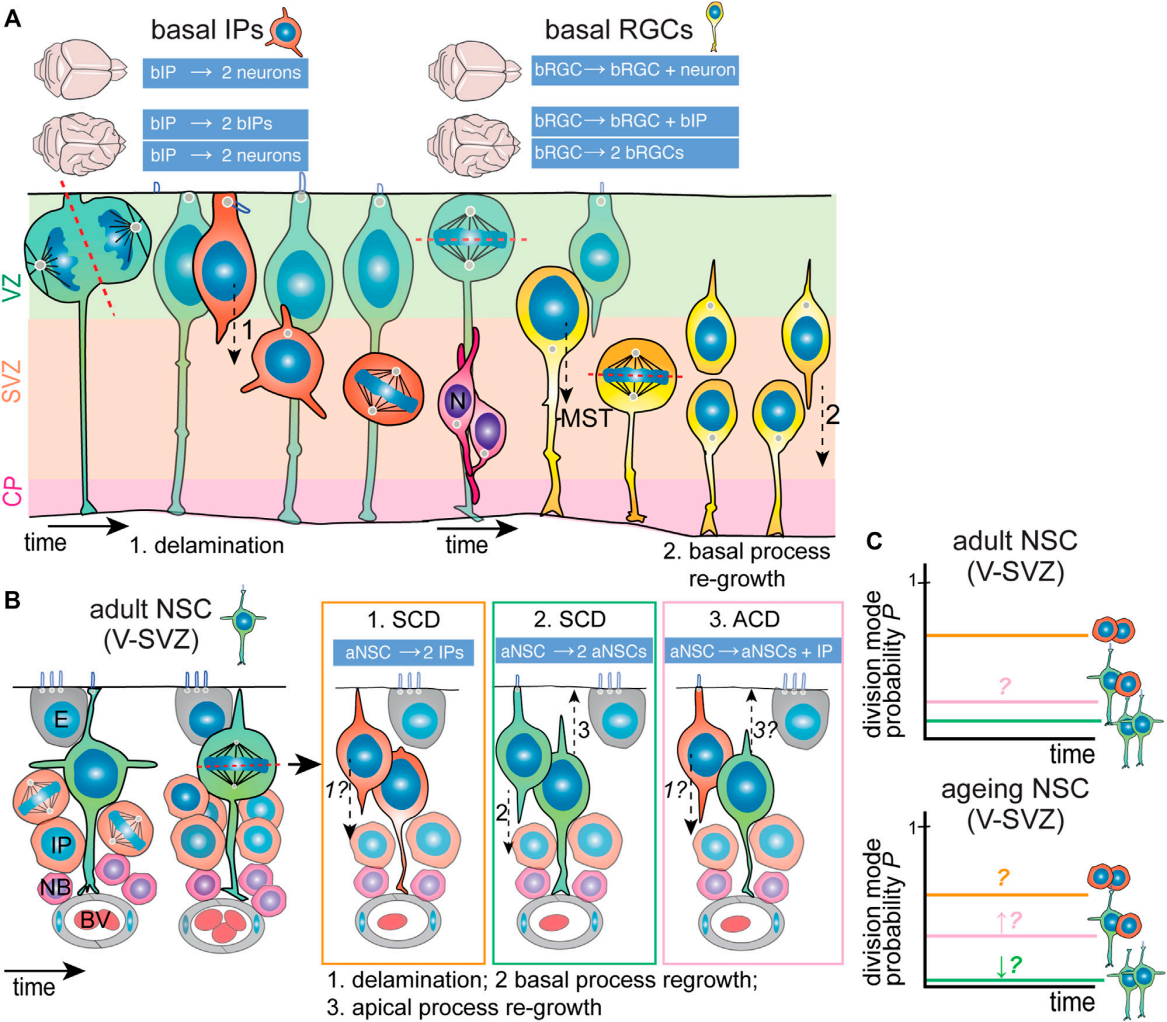
Division modes used by neocortex embryonic IPs and adult V-SVZ NSCs. **(A)** Basal IPs (orange; left) are born from asymmetric RGC divisions. Once born, bIPs delaminate from the ventricular zone (VZ, light green) and migrate basally. Here, they are unpolarized and divide with random spindle orientation. Depending on the species and extent of lissencephaly/gyrencephaly, bIPs generate mainly neurons (magenta) directly (lissencephalic) or can also undergo self-renewing divisions (gyrencephalic brains). Basal RGCs (yellow; right) are born from horizontal RGC divisions. Once born, they relocate to the inner and outer subventricular zones (SVZ, light orange) where to undergo mainly horizontal divisions, preceded by mitotic somal translocation (MST). Depending on the species and extent of lissencephaly/gyrencephaly, they divide asymmetrically generating one self-renewing and one differentiating daughter cell (lissencephalic) that migrates to the cortical plate/neuronal layers (CP, light purple) or can also undergo self-renewing divisions (gyrencephalic brains). In the latter case, the apical-most daughter cell can re-grow a basal process. **(B)** Adult NSCs (green) in the ventricular-subventricular zone (V-SVZ) are positioned within a specialized niche. They are polarized and have a small apical domain neighboring the lateral ventricle. Once activated, they appear to undergo mainly horizontal divisions, and can generate two IPs (orange; option 1); expand through symmetric division (green; option 2) or (rarely) undergo asymmetric division (pink; option 3). In turn, IPs generate neuroblasts (magenta) that also amplify and migrate to the olfactory bulb. It appears that aNSC daughter cells possess the potential to re-grow apical (3) and basal (2) processes. Possibilities regarding delamination from the ventricular zone and process regrowth are indicated with italics and question marks. **(C)** The current models of division mode usage by aNSCs (top graph). Presumably, the main division mode is symmetric differentiative division (∼70%–80%). The presence of asymmetric divisions is controversial due to conflicting data, though it has been proposed that asymmetric division increases during ageing (bottom graph). ACD, asymmetric cell division; aNSC, adult neural stem cell; bIP, basal intermediate progenitor; bRGC, basal radial glial cell; BV, blood vessel; E, ependymal cell; IP, intermediate progenitor/transient amplifying progenitor; N, neuron; NB, neuroblast; NSC, neural stem cell; SCD, symmetric cell division; V-SVZ, ventricular-subventricular zone.

Generally, two types of BPs can be distinguished that differ in cell architecture and proliferative potential: 1) low-proliferative basal intermediate progenitors (bIPs), which are not tethered to the pial surface by a basal process and have a multipolar morphology (Figure 4A, left), and 2) highly-proliferative basal radial glia (bRGCs, also termed outer radial glia), which retain the basal process and radial architecture (Figure 4A, right). While the cause for difference in proliferative ability between these two subtypes is still an open question, it appears that the retention of the basal process, known to be of critical importance in apical NPC stemness, is a key factor. Though bRGCs constitute a small percentage of total BPs in the mouse neocortex, they are much more abundant in the embryonic brain of gyrencephalic species (Kalebic et al., 2019). It is now believed that bRGCs are indeed the cellular basis of neocortical folding. Efforts from different research teams have identified several ape-specific or human-specific genes that are able to induce the generation of bRGCs and tissue folding when expressed in mice embryonic brains (Florio et al., 2015; Ju et al., 2016).

bRGCs are born from apical NPC divisions, and thus can inherit an apical process that needs to be disassembled for their migration to the OSVZ (Tavano et al., 2018). Interestingly, research seems to indicate that the mechanisms underlying delamination of bRGCs and differentiating neurons are shared to an extent. For instance, the centrosomal protein Akna plays a role in delamination of both cell populations, purportedly by mobilizing microtubules away from junctional complexes (Camargo Ortega et al., 2019). Similarly, the microtubule-associated Lzts1 promotes bRGCs and neuron production by inducing apical constriction and inducing oblique divisions in apical NPCs of mice and ferrets (Kawaue et al., 2019). Recently, the gene LIS1, which codes for a dynein regulator and mutations in which cause lissencephaly, has also been shown to be important for bRGC production, as well as for neuronal migration (Penisson et al., 2022). These commonalities between differentiating neurons and delaminating bRGC further illustrate the intermediate character of bRGCs.

Still, bRGCs show certain distinct behaviors that distinguish them from bIPs. They typically divide with a horizontal division with the basal process being inherited by the basal-most daughter cell (Figure 4A). In mice, bRGCs divide mainly asymmetrically generating one bRGC daughter and a differentiating daughter cell. In contrast, in gyrencephalic brains, bRGCs have higher self-renewing capacity and can divide symmetrically into two bRGCs or asymmetrically into one bRGC and a bIP [Figure 4A (Wang et al., 2011; Betizeau et al., 2013; LaMonica et al., 2013; Gertz et al., 2014)]. The actin cytoskeleton is important for regulation of the length and direction of the basal processes, which in human bRGCs is regulated by the Rho GTPases Rac1 and Cdc42 activity downstream of mTor signaling (Andrews et al., 2020). Prior to mitosis, bRGCs undergo a rapid movement of their cell body to the OSVZ, which is termed Mitotic Somal Translocation (MTS). Similarly to interkinetic nuclear migration, MTS is coordinated in time with mitosis but functionally independent. Mechanistically, MTS is regulated by the actomyosin cytoskeleton (Ostrem et al., 2014; Kawaue et al., 2019; Andrews et al., 2020). The precise biological significance of MTS has still not been characterized, but authors have speculated that MTS might serve to reduce tissue crowding. Intriguingly, MTS is a cell-autonomous mechanism as it occurs even in dissociated bRGCs (Ostrem et al., 2014). Experiments have shown that inhibition of MTS does not directly affect cell fate, but whether it influences the long-term proliferative potential of individual bRGCs needs further investigation.

Collectively, the proliferative capacity of individual BPs is highly heterogeneous (Pfeiffer et al., 2016), yet the basis of this heterogeneity is not fully understood. Single-cell RNA-seq expression profiling has uncovered some previously unidentified subtypes of BPs in human embryonic neocortex (Pebworth et al., 2021). Moreover, live imaging studies have revealed distinct characteristic morphotypes in gyrencephalic species (Betizeau et al., 2013; Kalebic et al., 2019; Pebworth et al., 2021). Strikingly, further research has shown that BP morphology has a strong influence on its proliferative ability (Betizeau et al., 2013; Kalebic et al., 2019). Aside from the basal process of bRGCs, both bRGCs and bIPs have small filiform protrusions of the cell body called lamellate expansions, and the number and length of these expansions positively correlate with proliferative potential. Moreover, these are present in human and ferret BPs, but not in the less proliferative mice BPs (Kalebic et al., 2019). A possible mechanistic basis underlying this correlation between cell body protrusions and proliferation might be that lamellate expansions mediate the reception of extracellular signals. For instance, integrin signaling through these protrusions has been demonstrated to support BP proliferation (Kalebic et al., 2019). In agreement with this, RNA-seq profiling of human BPs has also shown particular enrichment of extracellular matrix (ECM) related genes (Pollen et al., 2015). Lamellate expansions also mediate Notch signaling between BPs and apical RGCs (Nelson et al., 2013). Other signaling pathways known to influence BP proliferation are Sonic hedgehog, which promotes BP production both by stimulating apical RGC proliferation and BP self-renewal (Hou et al., 2021), and the Hippo pathway (Kostic et al., 2019). Conversely, Robo/Slit signaling promotes direct neurogenesis (Cárdenas et al., 2018). The identification of different BP subtypes open the possibility that signaling pathways influence specific subpopulations of BPs differently. Further efforts into characterizing the biological differences between BP subtypes and their relation to potential differences in proliferative ability will hopefully bring some answers in the near future.

As many advances as have been made in the knowledge of the morphological and transcriptional features that govern BP biology, the cell division symmetry or asymmetry mechanisms in these cells have not been uncovered. Furthermore, it is not known whether they show also show specific (spatiotemporally controlled) probability distribution of division modes. BPs lack signaling from the ventricle, apical membrane and classic apicobasal polarity and lateral junctions, all of which are important factors in establishing symmetry in apical NPCs divisions. It will be interesting to see to what extent these differences reflect on the mechanism of division mode selection.

## 6 How is Neural Progenitor Division Mode Regulated in the Adult Nervous System?

Towards the end of embryonic mouse neurogenesis, a subset of RGCs slows their cell cycle and turns into adult NSCs (Fuentealba et al., 2015; Furutachi et al., 2015; Berg et al., 2019). Whereas mammalian species retain just a limited number of NSC niches where adult neurogenesis occurs, other vertebrates such as zebrafish show much more widespread neurogenesis (Labusch et al., 2020). However, adult NSCs (aNSCs) from different vertebrate species have in common that they are largely quiescent, and divide only rarely. When activated, the aNSCs generate intermediate progenitors that in turn undergo several divisions to increase neuronal output. The adult NSC zones in mammals are the ventricular-subventricular zone (V-SVZ, also called subependymal zone SEZ) of the lateral ventricle, and the subgranular zone (SGZ) in the hippocampal dentate gyrus in the hippocampus. The SEZ generates olfactory neurons and oligodendrocytes, with the long-term NSCs being mainly quiescent and activated NSCs producing transient amplifying progenitors and neuroblasts that are neurogenic. While aNSCs have certain morphological properties, such as apicobasal polarity, in common with embryonic progenitors, the adult NSC niche is an important regulator of NSC divisions and differentiation and the lineages produced are more restricted than that of embryonic NPCs [reviewed by (Obernier and Alvarez-Buylla, 2019)].

Evidence regarding aNSC division modes is sparse, as the tissue is less amenable to live imaging experiments and divisions of the *bona fide* stem cells are rare. Fortunately, elegant lineage tracing approaches and recent advances in microscopy have provided insights in aNSC division modes and their regulation (Figure 4B). Several studies using time-lapse imaging and lineage tracing methods have now shown that in the mammalian SEZ, asymmetric division of aNSCs hardly occurs. Instead, stochastic selection of either symmetric proliferative or differentiative divisions, with higher probability of the latter, have been observed [Figures 1E, 4B,C (Basak et al., 2018; Obernier et al., 2018)]. From these studies, maintenance of quiescent NSCs (qNSCs) during the production of neurons was proposed to occur at the population level (population asymmetry), rather than invariant division asymmetry (Figure 1F) at the individual level. In this study, an important role was proposed for strict regulation and qNSC occupation in the niche similar to qNSC maintenance in the intestine. aNSCs were found to be able to return to quiescence to maintain the number of qNSCs per niche (Basak et al., 2018). Computational modeling based on lineage-tracing data comparing young and old mice showed that asymmetric division might actually be more prevalent than previously proposed (Figure 4C) (Bast et al., 2018). This modeling also predicted that over time, the probability of an aNSC to undergo asymmetric divisions (Figure 4C) increases and the frequency of qNSC activation and inactivation is decreased, leading to NSC progeny being less mature and with fewer activated NSC at each timepoint (Figure 4C) (Bast et al., 2018). These findings indicate that for the SEZ, data on the division modes used are contradicting and that next to population asymmetry, asymmetric divisions may also play a role.

In contrast, recent work on the SGZ in the adult hippocampus have indicated that the aNSC division modes are more diverse and are similar to that of embryonic NSCs (Pilz et al., 2018; Bottes et al., 2021). Time-lapse imaging and computational modeling showed that hippocampal aNSCs undergo division mode switches over time consisting of initial symmetric self-renewing divisions, followed by increasing probabilities for asymmetric and symmetric terminal subsequent divisions (Pilz et al., 2018; Bottes et al., 2021). Lineage-tracing in the adult zebrafish dorsal telencephalon demonstrated a subpopulation of deeply quiescent cells that divide asymmetrically, generating a more active pool of NSCs that choose their division mode stochastically (Than-Trong et al., 2020). Taken together, studies from several vertebrate organisms indicate that adult NSCs show specific hierarchies, with possible presence of an asymmetrically dividing deeply quiescent reservoir NSCs and a more actively cycling pool of activated NSCs and progenitors. However, evidence is conflicting as to whether activated NSCs and progenitors show consistent population asymmetry through symmetric divisions or whether asymmetric division is also of significance (Figure 4C, top panel).

Even though we now have some insight into the division modes used by aNSCs, very little is known about the molecular mechanisms, and how these are similar or different from embryonic NSCs. Recent studies have provided some clues (Figure 4B, right panels). Time-lapse imaging of aNSCs in mouse SEZ slices were shown to retain the basal process during mitosis, with the non-inheriting daughter cell able to regrow a basal process (Figure 4B) (Obernier et al., 2018) similar to early symmetric embryonic NPCs (Shitamukai et al., 2011; Fujita et al., 2020). In contrast to its effect on embryonic NPCs, spindle randomization in the SEZ through acute overexpression of Insc does not affect the numbers of aNSCs and neuroblasts in mouse SEZ (Falk et al., 2017). Similar to embryonic NSC divisions, asymmetric segregation of signaling components and fate determinants could play a role in aNSC as well. For example, asymmetric segregation of Delta1 ligands has been observed in SEZ NSCs *in vitro* (Kawaguchi et al., 2013), but it is not yet clear whether this also happens *in vivo* and is related to specific daughter cell fates. A lateral endoplasmic reticulum (ER)-diffusion barrier has been demonstrated in both embryonic and adult hippocampal NSCs that mediates asymmetric segregation of damaged proteins into the differentiating daughter cell (Moore et al., 2015). Intriguingly, this barrier and asymmetric segregation of damaged proteins has been observed to weaken over age, which could contribute to the decreased functioning of aged aNSCs.

Similar to embryonic NPCs, epithelial properties such as apicobasal polarity is important for aNSCs. For example, recent work showed that the EMT factor Zeb1 is required to maintain hippocampal aNSC self-renewal and prevent premature differentiation. Here, Zeb1 maintains asymmetric division mode through regulation of the transcription factor Etv5, showing that specific TFs actively regulate division mode selection in the adult hippocampus (Gupta et al., 2021). The currently limited data suggest that segregation of apical/basal domains (Figure 4B) and asymmetric segregations of fate determining factors such as Notch ligands occur in aNSCs similar to embryonic NSCs (Obernier et al., 2018). However, seeing that aNSCs are very dependent on their niche and spend most of their time in quiescence, it is likely that the combination of mechanisms underlying the symmetry of their divisions is distinct from that in embryonic NPCs. Future work in species with higher levels of adult neurogenesis like the zebrafish, will hopefully provide more information on the specifics of division mode selection by aNSCs.

## 7 Conclusion and Outlook

In this review, we have summarized the current knowledge on how neural stem and progenitor division modes are determined, and how timely changes in division mode, and the proper balance between self-renewal and differentiation is key to brain development. Interestingly, recent studies regarding how cellular properties may be differentially regulated throughout development, show intriguing insights into the complexity of the molecular and cell biological mechanisms that underlie asymmetric and symmetric division. While individual mechanisms in asymmetric NSC division are reasonably well understood, an integrated view of the subtleties that go with gradual developmental changes in division mode outcomes is still far away. Moreover, we are just starting to understand how individual stem and progenitor cells determine their life path based on a combination of intrinsic and extrinsic input, including deterministic processes, such as fate determinant inheritance and stochastic processes such as gene expression levels that in turn influence cellular properties.

Based on the currently available information, a number of relevant outstanding questions can be identified. For example, how are transcriptional changes during development coupled to the gradual changes in NPC morphology, organelle inheritance and division modes? Despite our knowledge on the general role of signaling pathways in regulation of the proliferation versus differentiation balance, we know very little about the details at the individual cell level. For instance, what are the combinatorial effects of different signaling pathways affect division mode selection? What is the effect of cell cycle stage-specific activation states of signaling pathways on individual NPC division modes? Recent studies have identified intriguing links between centriole asymmetry and signaling in embryonic NPCs. It would be very interesting to explore this link further and to understand more on how the compensatory mechanisms to overcome centriole age differences are regulated over time transcriptionally. Furthermore, the role of centriole age asymmetries in aNSC lineage progression is completely unknown. Ultimately, NPC division mode outcomes depend on combinations of different mechanisms. If more hidden asymmetries and the weight of each deterministic factor is known, it is interesting to explore how well individual NPC division outcomes could be predicted using computational models.

As we have discussed, the mechanisms regulating division mode in adult NSCs are currently underexplored. Here, it would be of use to assess the differences and similarities in the mechanisms determining the symmetry of division in embryonic versus adult NSCs further. For instance, considering the prevalence of symmetric divisions in adult germinal zones such as the SEZ, is regrowth of apical and basal domains also more prevalent? How are embryonic NSC initially selected as adult NSCs and which mechanisms (for instance transcriptional and epigenetic changes) underlies their slowing of the cell cycle? Are individual quiescent aNSCs maintained through invariant asymmetric divisions, and which mechanisms are involved in mediating such asymmetric fate outcomes? Furthermore, as in mammals, regeneration and repair of nervous system damage is limited, it would be valuable to explore further how neurogenesis in vertebrate species such as fishes with higher capacity for neuronal regeneration is regulated and how regulation of division modes is involved in regeneration.

To address these questions, specific challenges remain in connecting the individual fate-determining factors, asymmetries and processes and their interactions to find how the combination and weight of each deterministic and stochastic mechanism influences the division mode of each NPC type. Here, the recent advances in and increasing number of published reports on single-cell analysis [like single-cell genomics and proteomics, e.g., (Schier, 2020)] studies of NPCs in multiple life-stages and species, could play a key role to unravel those subtle hidden asymmetries. Moreover, mathematical modelling and simulations have proven extremely valuable in understanding the connection between specific fate-determining mechanisms and stem cell choices. In combination with more classic approaches such as lineage tracing and time-lapse microscopy (VanHorn and Morris, 2020), these approaches should lead us to integrated and robust models of NPC division outcomes in different life stages and distinct species.
